# Mental Health Professionals’ Attitudes Towards the Network Theory of Mental Disorders

**DOI:** 10.32872/cpe.17941

**Published:** 2026-02-27

**Authors:** Lea Schumacher, Levente Kriston

**Affiliations:** 1 Department of Medical Psychology, University Medical Center Hamburg Eppendorf, Hamburg, Germany; Friedrich-Alexander-Universität Erlangen-Nürnberg, Erlangen, Germany

**Keywords:** network theory of mental disorders, mental health professionals, models of psychopathology, attitudes, survey

## Abstract

**Background:**

The network theory describes mental disorders as a network of interacting symptoms. While most research on the network theory is based on network analyses of symptom data, little is known about mental health professionals´ attitudes towards this theory. Clinical expertise could offer a valuable additional perspective on the validity of the theory and its applications to clinical practice.

**Method:**

Mental health professionals rated their agreement with propositions of the network theory regarding the phenomenology, aetiology, and treatment of mental disorders in an online survey. Further, the acceptability and appropriateness of possible applications were evaluated. We calculated descriptive statistics and examined associated factors with regression analyses.

**Results:**

The participating psychotherapists (*n* = 183), specialized physicians (*n* = 45), and clinical psychologists (*n* = 29, total *n* = 257) largely agreed with the network theory’s propositions regarding the phenomenology of mental disorders and treatment effects. Appraisal of the network theory regarding the aetiology of mental disorders, regarding important treatment targets, and regarding acceptability and appropriateness of possible applications was mixed. A theoretical background in cognitive behavioural therapy and previous knowledge of the network theory were associated with a stronger agreement in most domains.

**Conclusions:**

The fundamental assumptions of the network approach seem to resonate with mental health professionals, while the consequences for the diagnosis and treatment of mental disorders were questioned. Our findings indicate that the general conceptualization of mental disorders as symptom networks seems to align with mental health professionals’ perceptions but, at the same time, emphasizes the novelty and limited specificity of the theory’s implications for clinical practice.

## Highlights

- Clinicians could provide a novel perspective on the validity of the network theory.
- Most clinicians supported the conceptualization of mental disorders as symptom networks.
- The implications of the network approach regarding treatment targets were considered more critical.
- Core claims of the network theory were supported, but consequences for treatment were questioned.

With accumulating critique for traditional classification systems of mental disorders, the field of clinical psychology and psychiatry has been experiencing a movement towards reconsidering the conceptualization and classification of psychopathology (Eaton et al., 2023; Leucht et al., 2024; Rief et al., 2023). Various new models and frameworks were proposed (e.g., Insel et al., 2010; Kotov et al., 2017; Scheffer et al., 2024b). One prominent new approach, the network approach, describes mental disorders as a network of mutually interacting symptoms (Borsboom, 2017; Borsboom & Cramer, 2013; Scheffer et al., 2024b). Specifically, the network theory proposes that mental health problems develop and persist through causal relations between individual symptoms. While the occurrence of a specific symptom might be originally caused by “external” factors such as stressful life events or neurological problems, reciprocal interactions and feedback loops between symptoms are thought to lead to the persistence of mental disorders (Borsboom, 2017; Cramer et al., 2016; Scheffer et al., 2024b). This conceptualization contrasts to the disease model of mental disorders which posits that symptoms co-occur because of a common underlying cause (Borsboom & Cramer, 2013).

The number of empirical studies that investigate mental disorders from a network perspective is rapidly rising (Fried et al., 2017; Robinaugh et al., 2020; Schumacher et al., 2024) and applications of this approach to clinical practice have been frequently discussed (Blanchard & Heeren, 2022; McNally, 2016; Rodebaugh et al., 2020; Westhoff et al., 2024). It is often suggested that symptom networks can be used for personalized treatment planning, for example, by integrating it in case conceptualization (Burger, Ralph-Nearman, et al., 2022; von Klipstein et al., 2020) or by choosing a treatment target for the individual person (Levinson et al., 2021). According to the network theory, treatment should target 1) external factors that cause specific symptoms, 2) specific symptoms that relate to many other symptoms, i.e., are central in a given network or 3) strong symptom interactions so that the occurrence of individual symptoms ceases to cause other symptoms (Borsboom, 2017).

Empirical evidence for the claims of the network theory is so far rather mixed. For example, empirical evidence for the importance of strongly connected symptoms or for the proposition that symptom networks become less connected through successful treatment is still lacking (Lee et al., 2024; Rodebaugh et al., 2018; Schumacher et al., 2023; Spiller et al., 2020). Further, network analysis faces several methodological challenges, which make the explicit testing of this theory through modelling symptom data difficult (Bastiaansen et al., 2020; Bringmann et al., 2022; Schumacher et al., 2024; Siepe et al., 2025). An important source of evidence that could support, refine, or challenge the network theory of mental disorder is the clinical expertise of mental health professionals. Mental health professionals accumulate information on symptoms of their patients in their daily practice and are, thus, a potential source of information about the phenomenology, aetiology, and treatment of mental disorders. Further, while the network approach to psychopathology is extensively discussed within the scientific community, exchange between clinicians and researchers allows knowledge transfer in both directions and could provide valuable information on how the network approach could be successfully applied to clinical practice (Rodebaugh et al., 2020).

A few studies evaluated mental health professionals’ assessments of the feasibility, acceptability and utility of the network approach in clinical practice. The data collection necessary for constructing a personalized symptom network was rated as feasible and acceptable by therapists and patients and therapists stated that symptom networks were helpful and provided new insights (Frumkin et al., 2021; Kroeze et al., 2017; Scholten et al., 2022, 2025). In another study, therapists indicated interest in using personalized symptom networks for case conceptualization and psychoeducation, but few discussed the provided symptom networks with their patients (Hall et al., 2023). Further, the network’s interpretation was perceived as challenging (Scholten et al., 2022) and some therapists did not agree that networks would provide additional information for their clinical work (Frumkin et al., 2021). While these studies provide initial insights, they were based on interviews with less than 30 therapists per study. Additionally, they mainly focussed on the application of the network approach to clinical practice, i.e. did not assess attitudes towards the theory. Therefore, a large scale, systematic assessment of the attitudes of mental health professionals regarding the network theory of mental disorders is needed. In the present study, we aimed to investigate (a) to which degree mental health professionals agree with propositions of the network theory regarding the phenomenology, aetiology and treatment of mental disorders, and (b) how acceptable and appropriate they rate potential applications of the network approach in clinical practice.

## Method

### Procedure

We created an online survey to assess attitudes towards the network theory of mental disorders. The target respondents were mental health professionals, including clinical psychologists (persons with a university degree in psychology without additional training, but working in the field of mental health), psychotherapists (either graduated or in training), and physicians specialized in psychiatry and psychotherapy, psychosomatic medicine, neurology, or child and adolescent psychiatry (either graduated or in training). In Germany, mental health treatments are mostly provided by these professions. All three professions require a university degree in psychology or medicine, and psychotherapists and specialized physicians additionally receive extensive training in psychotherapy and/or their respective field of specialization.

The survey was made available online in English and in German using LimeSurvey (LimeSurvey GmbH, 2024). It was distributed by reaching out to 111 international mental health related associations (e.g. all national organizations of the *International Association for Analytical Psychology* and of the *European Association for Behavioural and Cognitive Therapies*), to 137 German mental health related associations (e.g., all regional chambers of psychotherapists and regional and national psychotherapeutic and psychiatric organizations), and to 77 large European psychiatry clinics. Further, the survey was shared on social media (X, LinkedIn, Instagram) and by reaching out to colleagues. Previous knowledge of the network theory was not required and participants received no incentives.

Before proceeding with the survey, it was pointed out that participation is voluntary and the survey would take a maximum of 20 minutes. All participants had to consent to the privacy statement (only personally non-identifiable data were collected) and to take part in this study. The data collection started in October 2023, and the last included response was recorded in June 2024. We aimed to achieve a sample size of 250 respondents, as simulations indicated that sufficient precision for descriptive analyses can be achieved with this sample size. The study design was preregistered on the open science framework: https://osf.io/g7zy3. We did not specify specific hypotheses. The study was approved by the local psychological ethics committee of the centre of psychological medicine at the University Medical Centre Hamburg Eppendorf (LPEK-0607).

### Questionnaire

The questionnaire was based on previously published presentations of the network theory by Borsboom and Cramer (Borsboom, 2017; Borsboom & Cramer, 2013). It was first developed in English and then translated to German with the help of ChatGPT (OpenAI, 2023). The first version was tested and adjusted for better comprehensibility with the help of two psychotherapists. Several clinical researchers assessed the final implemented version for mistakes and intelligibility.

The survey included 48 questions in total. Eight questions assessed demographic variables. Subsequently, a description of the network theory of mental disorders was shown. It was described how the network theory explains the development and persistence of psychological symptoms and what consequences this has for the treatment of mental disorders. The network approach was contrasted with the disease model of psychopathology, which states that symptoms share a common underlying disorder. Both models were described using the example of depression and depicted with corresponding diagrams (see Supplementary Materials [Schumacher & Kriston, 2025S] for the full description). Participants were asked if they knew about the network theory and if they had knowingly used a treatment planning tool or an intervention based on the network approach before. Then, participants were asked how strongly they agree or disagree with specific propositions of this theory. These propositions related to three domains: the phenomenology, the aetiology, and the treatment of mental disorders. All items referred to mental disorders or symptoms in general and not to a specific mental health problem or disorder. One statement related to the overall agreement with the network theory and four statements relating to the disease model were also incorporated.

Additionally, we were interested in the acceptability and appropriateness of possible applications of the network approach to clinical practice. Acceptability and appropriateness were defined as important outcomes for successful implementation of new practices (Proctor et al., 2011). We asked for the level of agreement with the acceptability and appropriateness of specific applications, mainly relating to the use of symptom networks for treatment planning and outcome monitoring. Wording was inspired by a validated measure of acceptability (“I would like to use...”) and appropriateness (“It seems fitting to use…”, Weiner et al., 2017). Finally, we assessed the extent that mental health professionals already implicitly implemented the network approach in their practice, i.e. if they previously considered symptom interactions in their practice. The level of agreement for each proposition could be answered on a seven-point Likert scale from “strongly disagree” (-3) to “strongly agree” (3).

Participants could also indicate if they would like to use tools or techniques based on the network approach in any other way in their practice and give any other comment in two open text questions. Two questions were included to test for automated responding. The complete questionnaire can be found in the Supplementary Materials.

### Measurement Properties of the Questionnaire

To evaluate if mean scores for the agreement in each domain can be calculated, we assessed the measurement properties of the questionnaire. More specifically, we used confirmatory factor analyses to assess the fit of one-factor models for measuring agreement with the network theory regarding the phenomenology, aetiology, and treatment of mental disorders and the acceptability and appropriateness of possible applications. An acceptable to good fit was shown for measuring agreement with the network theory’s propositions on the phenomenology and aetiology of mental disorders and for measuring acceptability and appropriateness. The one-factor model for agreement with the network theory’s propositions regarding the treatment of mental disorders showed poor fit. Here, a two-factor model showed good fit. One factor indicated agreement with the network theory regarding treatment effects, e.g. that effective treatment leads to a reduction in symptom interactions, and the other factor measured agreement with treatment targets as proposed by the theory, e.g. that treatment should target individual symptoms and their interaction. Thus, a mean score was computed for agreement with the network theory’s propositions regarding the phenomenology and aetiology of mental disorders, treatment targets, treatment effects, and for the acceptability and appropriateness of possible applications. Previous considerations of symptom interactions in practice can be interpreted as formative indicators of a composite latent variable, thus, a mean score was also computed for this domain. The mean score was also computed when the response was missing for one item of the respective domain. The complete psychometric evaluation of the questionnaire is reported in the Supplementary Materials.

### Statistical Analysis

Data from all respondents that answered at least one question regarding the network theory of mental disorders were included. Data were excluded if the respondent indicated no mental health profession or answered both attentiveness checks wrong. The main outcome was descriptive statistics for the agreement with propositions of the network theory and the acceptability and appropriateness of possible applications. For each statement, we evaluated the level of agreement using mean scores, standard deviations, and ranges. Further, we used Bayesian linear regression analyses to assess which variables were associated with agreement in each domain of the network theory, the acceptability, appropriateness of its application, and previous considerations of symptom interactions in practice. First, age, being in training, mental health profession, work experience, and theoretical background were entered as predictors. Second, we added previous knowledge of the network theory and previous use of a treatment planning tool or intervention based on the network approach as predictors. We used Markow-Chain-Monte-Carlo sampling with weakly informative priors and 40,000 iterations to estimate the regression model with brms (Bürkner, 2017) in R 4.3.3 (R Core Team, 2013). Data is openly available (https://osf.io/zan2q/files).

## Results

### Sample Description

While the online survey was accessed 356 times, 265 persons answered at least one question regarding the network theory. Data was excluded for five persons who answered both attentiveness checks wrong, for one person who answered “neither agree nor disagree” for all questions, and for two persons who indicated that they only work as scientists. Demographic information of all 257 included participants is displayed in Table 1.

**Table 1:** Demographic Information

Variable	*N* (relative frequency)
Gender	
female	185 (72.0%)
male	67 (26.1%)
non-binary	1 (0.4%)
rather not say	4 (1.6%)
Age	
18-30 years	42 (16.3%)
31-50 years	126 (49.0%)
51-70 years	76 (29.6%)
> 70 years	12 (4.7%)
rather not say	1 (0.4%)
Profession^a^	
Clinical psychologist	29 (11.3%)
Specialized physician	45 (17.5%)
Psychotherapist	183 (71.2%)
Training Status	
No	180 (70.0%)
Yes	77 (30.0%)
Proportion Completed Training	
1-20%	10 (13.0%)
21-40%	10 (13.0%)
41-60%	13 (16.9%)
61-80%	18 (23.4%)
81-100%	26 (33.8%)
Theoretical Background^b^	
Cognitive behavioural	177 (68.9%)
Psychodynamic / psychoanalytic	83 (32.3%)
Other	50 (19.5%)

^a^Data missing for two respondents. ^b^48 participants indicate several theoretical backgrounds.

Most participants were female, between 31 and 50 years old, and from Germany. About one third of the sample was currently in training, and of these, the majority had completed at least 60% of their training. Nearly three-quarters of the sample were psychotherapists, 17.5% specialized physicians (specialization in child and adolescent psychiatry, psychiatry, psychosomatic medicine, and/or neurology) and 11.3% clinical psychologists. Nearly 70% of the mental health professionals indicated a background in cognitive behavioural therapy, 32.3% in psychodynamic or psychoanalytic treatment, and about 20% indicated a different theoretical background (48 participants indicated multiple theoretical backgrounds). The mental health professionals had a mean of 16 years of work experience, ranging from one to 50 years.

The majority of the sample did not hear about the network theory before (70.8%), never used this approach for treatment planning (93.0%) and never knowingly administered an interventions based on the network approach (84.8%). The majority of the participants filled out the survey in German (93.0%) and the mean completion time was 15.8 minutes. All questions related to the phenomenology, aetiology, and treatment of mental disorders were answered completely by 245 participants, and 236 participants also answered all questions regarding the acceptability and appropriateness of possible applications, i.e., finished the complete survey.

### Agreement With the Network Theory

**Figure 1:**
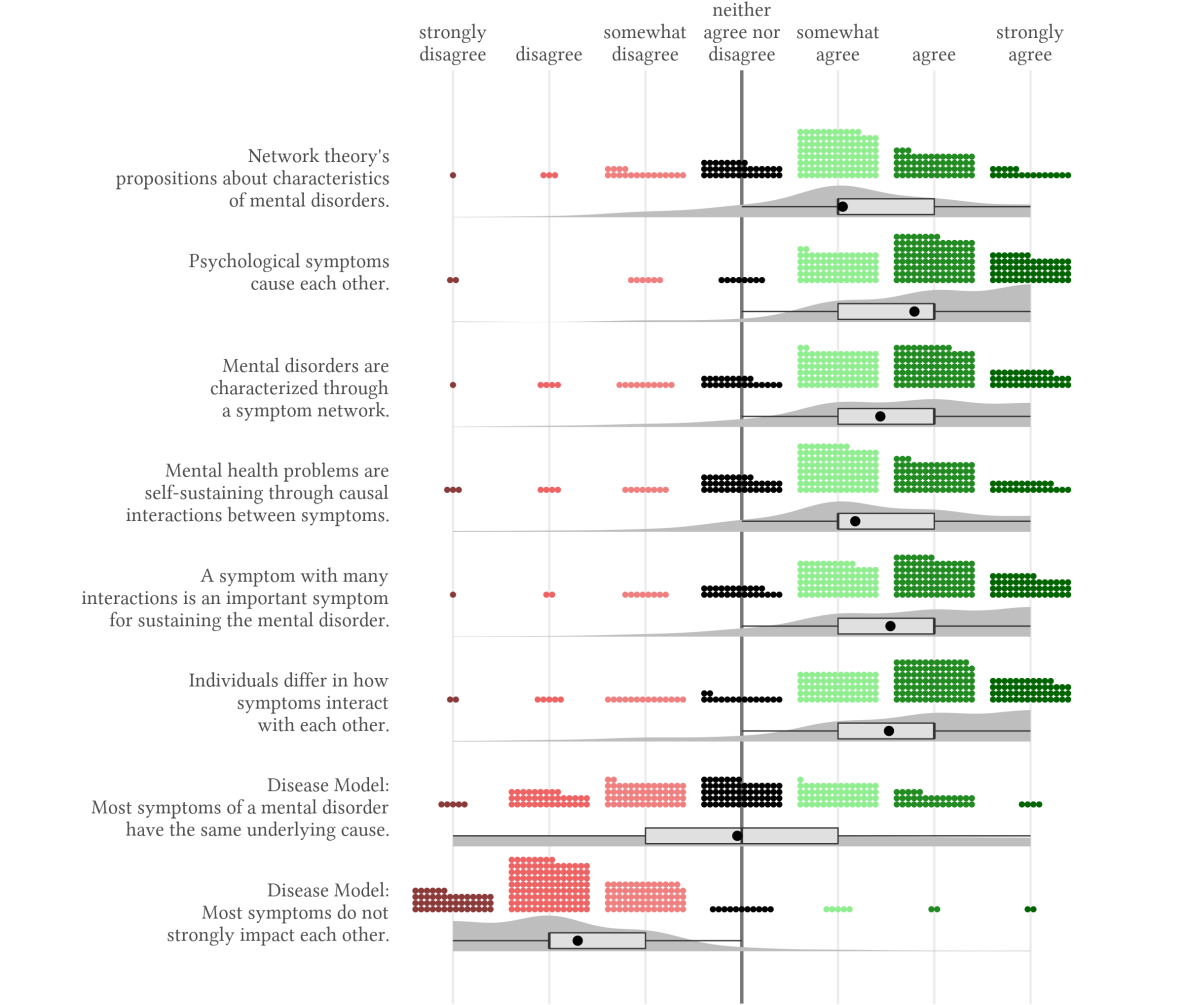
Agreement With Propositions of the Network Theory on the Phenomenology of Mental Disorders *Note.* The small coloured dots represent each response and the grey shaded area represents the estimated distribution of responses. The light grey box represents the interquartile range containing the middle 50% of the data. The black vertical line and the black dot show the median and the mean response for each statement.

**Figure 2:**
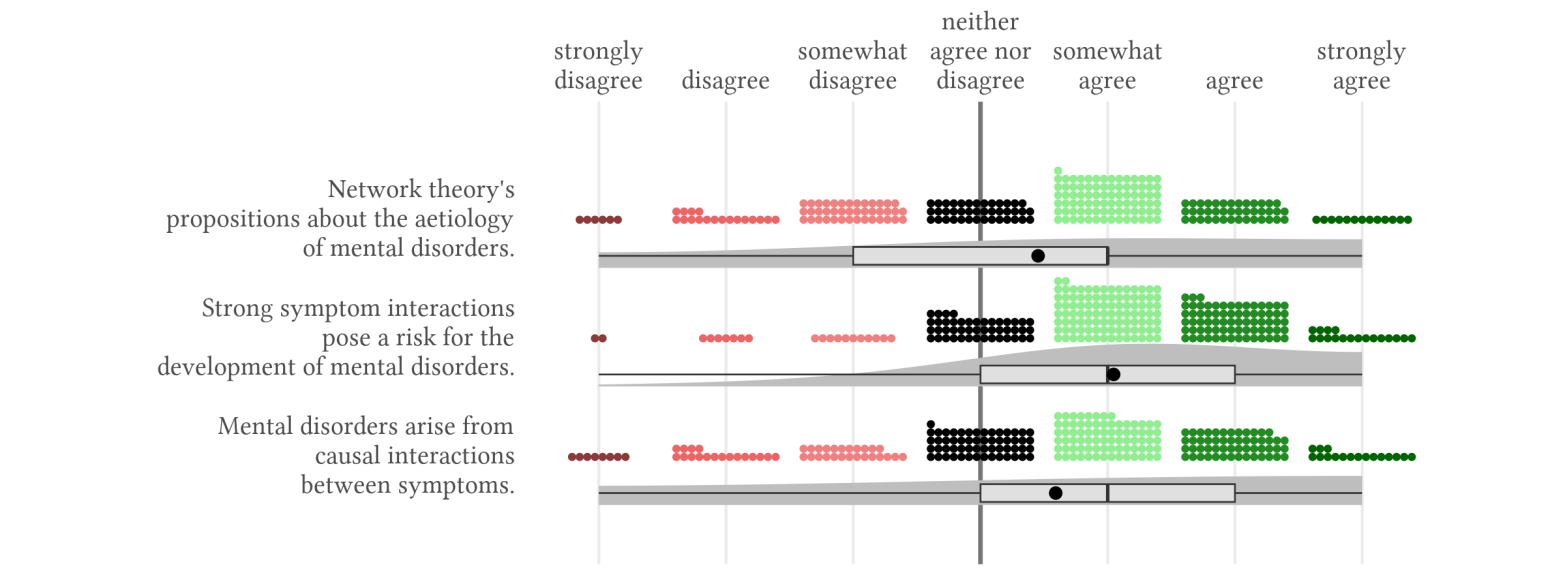
Agreement With Propositions of the Network Theory on the Aetiology of Mental Disorders *Note.* The small, coloured dots represent each response and the dark grey shaded area represents the estimated distribution of responses. The box represents the interquartile range containing the middle 50% of the data. The vertical black line and the black dot show the median and the mean response for each statement.

We found consistent agreement with propositions of the network theory regarding the phenomenology of mental disorders (Figure 1). The vast majority of participants rated all network theory’s propositions on the phenomenology at least moderately positively. The mean score for the agreement in this domain was 1.4, *SD* = 0.8 (scale ranges between -3 indicating strong disagreement, to 3 indicating strong agreement). Propositions of the disease model, this means the statements that most symptoms do not strongly impact each other and that most symptoms have the same underlying cause were, on average, evaluated negatively, *M* = -1.7, *SD* = 1.0 and *M* = -0.1, *SD* = 1.4. Participants strongly differed in their ratings of the network theory’s propositions regarding the aetiology of mental disorders, with a mean level of 0.7, *SD* = 1.1 (Figure 2). Still, we found that participants rated the proposition that strong symptom interactions pose a risk for the development of mental disorders moderately positively, *M* = 1.1, *SD* = 1.1.

**Figure 3:**
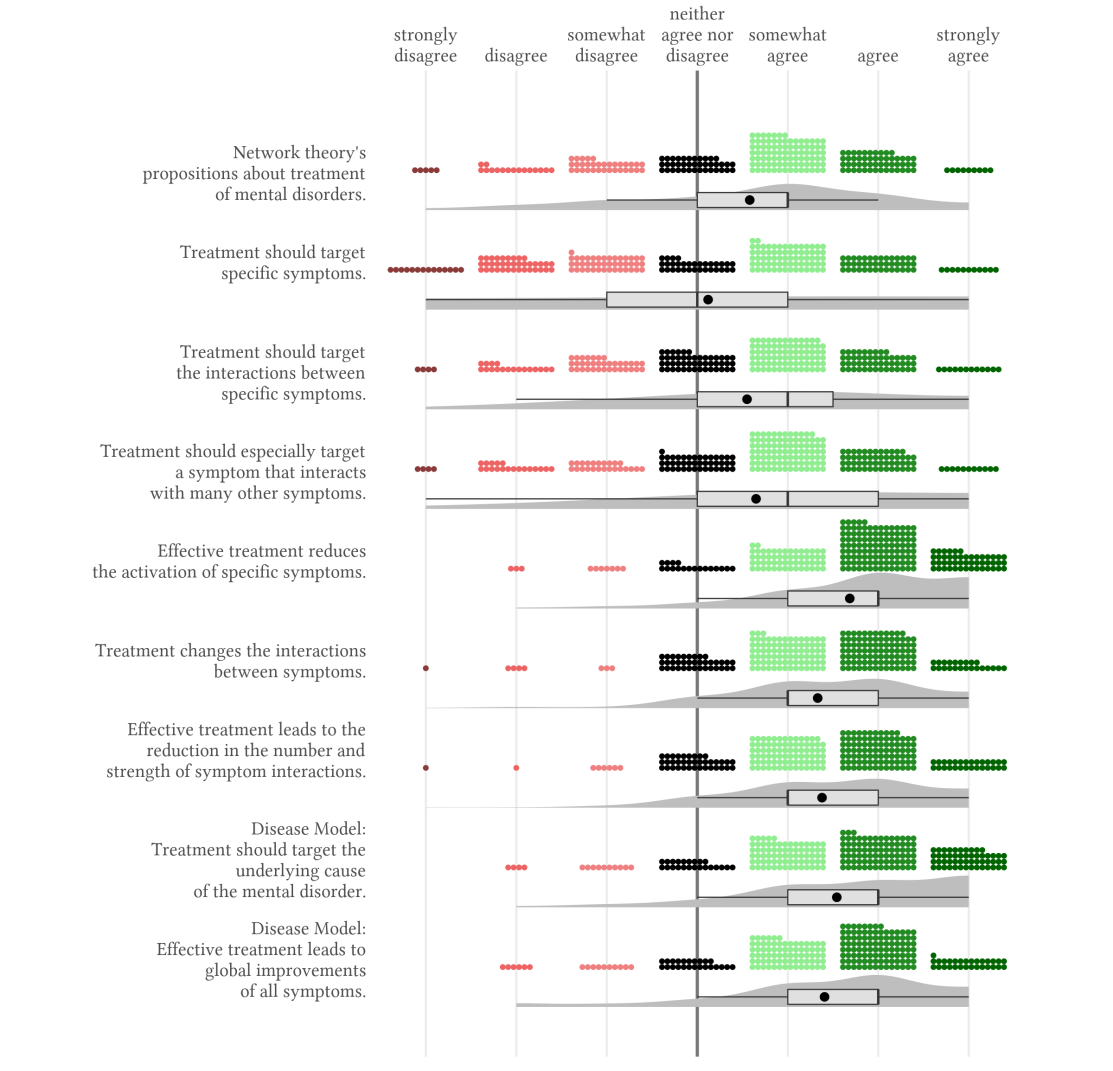
Agreement With Propositions of the Network Theory on the Treatment of Mental Disorders *Note.* The small, coloured dots represent each response and the dark grey shaded area represents the estimated distribution of responses. The box represents the interquartile range containing the middle 50% of the data. The vertical black line and the black dot show the median and the mean response for each statement.

Attitudes towards propositions of network theory regarding the treatment of mental disorders differed between two aspects of treatment (Figure 3). The evaluation of statements proposing that treatment targets should be selected according to the network theory was very mixed, *M* = 0.5, *SD* = 1.2. On the other hand, we found strong support for propositions regarding treatment effects, i.e., that treatment changes the activation of specific symptoms and symptom interactions, *M* = 1.5, *SD* = 0.8. Finally, we found strong agreement for the disease model’s proposition that treatment should target the underlying cause of the mental disorder and that effective treatment leads to global improvements across all symptoms, *M* = 1.5, *SD* = 1.1 and *M* = 1.41, *SD* = 1.09.

Agreement with the network theory’s propositions regarding the phenomenology and aetiology of mental disorders, treatment targets, and treatment effects correlated moderately with each other (see Table S1 in the Supplementary Materials).

### Variables Associated With Agreement With the Network Theory

Detailed results of the Bayesian linear regression analyses are presented in Table S2 in the Supplementary Materials. Age, gender, being in training, work experience, mental health profession, and theoretical background could explain agreement to the network theory’s propositions regarding the phenomenology, aetiology, treatment targets and treatment effects only to a limited extend as indicated by the Bayesian *R*^2^ ranging between .01 and .28. Still, a background in cognitive behavioural therapy was related to a more positive evaluation of the network theory regarding the phenomenology of mental disorders and treatment targets, *b* = 0.40 [95% credibility interval: 0.01 – 0.79] and *b* = 0.69 [0.20 – 1.18]. Having heard about the network theory and having used an intervention based on the network approach was related to stronger agreement with the network approach regarding the phenomenology of mental disorders, *b* = 0.36 [0.13, 0.60] and *b* = 0.43 [0.10, 0.76], and regarding important treatment targets, *b* = 0.33 [0.03, 0.64] and *b* = 0.55 [0.11, 0.98].

### Agreement With Possible Applications

We found that applications of the network approach to clinical practice were rated, on average, somewhat appropriate and slightly less acceptable, *M* = 1.2, *SD* = 1.1 and *M* = 0.9, *SD* = 1.3, respectively (see Figure S1 and S2 in the Supplementary Materials). However, especially the evaluation of acceptability varied to a large extend between the participants. Further, most participants considered symptom interactions in their clinical practice in general, *M* = 1.1, *SD* = 1.3, but few have used the symptom interactions to plan or monitor treatment, *M* = 0.1, *SD* = 1.7, and *M* = -0.1, *SD* = 1.7, respectively. While previous consideration of symptom interactions correlated moderately with the other domains, the correlation of appropriateness and acceptability with agreement in the other domains was rather high (see Table S1 in the Supplementary Materials).

### Variables Associated With Agreement With Possible Applications

Detailed results of the Bayesian linear regression analyses on associated factors for the acceptability and appropriateness of possible applications and the previous consideration of symptom interactions are displayed in Table S3 in the Supplementary Materials. Age, gender, being in training, work experience, mental health profession, and the theoretical background of the participants could explain 28.2 and 26.4% of the variance in the acceptability and appropriateness ratings of possible applications. Being a physician (in comparison to psychotherapists) and having a background in cognitive behavioural therapy was associated to a stronger agreement with the acceptability and appropriateness of possible applications of the network approach. Being between 31 years and 50 years old (compared to being 18 to 30 years old) and having a background in psychodynamic or psychoanalytic therapy was related to lower ratings of the acceptability and appropriateness. Additionally, we found that physicians (in comparison to psychotherapists) and participants with a background in cognitive behavioural therapy more strongly agreed to have considered symptom interactions in their work before.

### Additional Comments

Thirty-six participants (14.0%) provided additional comments on how the network approach could be used in clinical practice and 66 participants (25.7%) provided general additional comments. Most comments indicated a negative evaluation of the network theory. First, it was emphasized that the theory does not provide new insights, because considering symptom associations is already an established clinical practice. Second, it was commented that the focus on symptoms and their interactions is too superficial and disregards the underlying (biological) cause. Some participants also commented positively that the network approach provides a useful framework for an intuitive clinical practice.

## Discussion

To consider clinical expertise in evaluating the validity of the network theory of mental disorders and to assess the acceptability and appropriateness of possible applications, we conducted an online survey with mental health professionals. The participants largely agreed with the network theory in regard to the phenomenology of mental disorders, e.g., that mental disorders are characterized through a symptom network, and in regard to treatment effects, e.g., that symptom interactions change through treatment. The level of agreement was very mixed for the network theory’s propositions regarding the aetiology of mental disorders and treatment targets. While possible applications of the network approach to clinical practice were rated, on average, somewhat acceptable and appropriate, participants varied largely in their ratings. A theoretical background in cognitive behavioural therapy, being a specialized physician, and previous knowledge or experience with the network theory were associated with more positive ratings of the theory and its application. While many mental health professionals considered symptom interactions in their practice, few used them for treatment planning or monitoring. Further comments showed that some participants did not see any novelty in the network approach, and some note that the network theory disregards the underlying cause of symptoms.

The current study provides a new perspective on the validity of the network theory: the expertise of practicing mental health professionals. Interestingly, our findings align rather well with previous findings based on network analyses with symptom data. The general conceptualization of mental disorders as symptom networks and its meaning for treatment effects was supported, similar to studies on symptom data showing that symptoms are associated with each other, and that symptom interactions seem to change through treatment (Cramer et al., 2016; Fried et al., 2017; Fried & Nesse, 2015; Schumacher et al., 2023). At the same time, specific implications of the network conceptualization for treatment, i.e. defining specific symptoms or symptom interactions as treatment targets, were seen more critical. This aligns with findings based on cross-sectional network analyses indicating that network connectivity and change in central symptoms may not relate to treatment response (Lee et al., 2024; Rodebaugh et al., 2018; Spiller et al., 2020).

The inconsistency of agreeing with the importance of causal symptom interactions and disagreeing with consequences of this assumption for treatment, i.e. targeting these interactions, might reflect the novelty of the network approach. Up until now, major classification systems define symptoms as merely passive agents, indicative of an underlying disorder, and treatment guidelines hardly focus on specific symptoms and their interactions (American Psychiatric Association, 2013; Hofmann & Hayes, 2019; Kendler, 2019). This is in line with the strong agreement of mental health professionals for targeting the underlying cause of mental disorders, found in this study. While the network theory does not disregard “external” causes for individual symptoms, it shifts the attention to the problem-maintaining process, i.e. symptom interactions. For a person with strong positive symptom interactions, a small external trigger can lead to the occurrence of many additional symptoms (Cramer et al., 2016; Lunansky et al., 2025; Scheffer et al., 2024a, 2024b). If we assume that symptoms cause each other and that there is no common underlying cause for *all* symptoms, treatment has to address symptom interactions to lead to a resilient, healthy state (Lunansky et al., 2025; Scheffer et al., 2024a). Currently, the field might be transitioning away from the previously dominant paradigm of the disease model towards new approaches to the classification and conceptualization of mental disorders, possibly causing conflicting theoretical models and observations (Eaton et al., 2023; Kuhn, 1996; Rief et al., 2023).

Additionally, our somewhat contradicting findings might be related to the limited specificity of the network theory. While it describes a general framework for conceptualizing and treating mental disorders, specific consequences for diagnosis and treatment of (specific) mental disorders remain vague. Similarly, the network conceptualization might be suitable for some mental disorders but not for other disorders (Fried & Cramer, 2017).

### Application of the Network Theory

The network approach could potentially provide a framework for systematically considering symptom interactions in the diagnosis and treatment of mental disorders (von Klipstein et al., 2020). Person-specific symptom networks could provide a standardized basis for case conceptualization (Burger, Epskamp, et al., 2022; Vogel et al., 2025; von Klipstein et al., 2020) and might be used for psychoeducation and selection of treatment targets (Levinson et al., 2022; Meier et al., 2022). Further, the network approach is reflected in calls for process-based therapy, which focuses on targeting symptom maintaining processes (Hofmann & Hayes, 2019). However, similar to previous studies, we found rather mixed agreement with the acceptability and appropriateness of possible applications of the network approach. Methodological challenges for person-specific networks based on empirical symptom data remain, and guidelines for network construction are only slowly emerging (Mansueto et al., 2023; Schumacher et al., 2024; Vogel et al., 2025). Given the currently available methods, the interpretation of person-specific networks should be seen as mainly hypothesis generating (von Klipstein et al., 2020). It also remains difficult to derive direct treatment applications from a person-specific symptom network (Levinson et al., 2021). Given the juvenile state of current applications, mixed findings in regard to mental health professionals ratings of the acceptability and appropriateness of possible applications are not surprising. Before applications of the network theory should be established, more knowledge on the theory itself and on the specific methodologies to derive symptom networks is indispensable.

### Limitations and Future Research

Our results might not generalize to mental health professionals working in other countries, as the majority of the sample was living in Germany. Second, due to privacy reasons, we did not collect data on racial/ethnic identification, measures of income, and socioeconomic status; thus, the impact of these variables could not be assessed. Similarly, we did not assess which problems were typically treated by the included mental health professionals. Although all survey items related to mental disorders or symptoms in general, participants might have considered a specific disorder when answering the questions, possibly influencing their answers. Finally, mental health professionals rated the acceptability and appropriateness of hypothetical applications of the approach. Ratings might have differed for actual implementations, i.e., if mental health professionals would have actually used tools based on the network approach in their practice.

Future research needs to further assess the validity of the network theory of mental disorders. On the one hand, studies might analyse empirical symptom data, for example assessing how certain network structures relate to mental health (problems). Hereby, methodological challenges for network analyses and a match between theoretical assumptions and the statistical model should be considered. On the other hand, existing clinical expertise could be further considered, for example by conducting interviews with experienced mental health professionals. Existing clinical theories might be mapped onto the network conceptualization. It needs to be considered to what extent a thorough understanding of the network theory is needed to evaluate its validity and usefulness for clinical practice and if the network approach is applicable to all kinds of different mental health problems. Combining various sources of information, i.e. empirical symptom data and clinical expertise, will hopefully lead to a better understanding of mental disorders.

### Conclusion

We showed that mental health professionals agree with central claims of the network theory, however, most of them were hesitant in regard to the applications of this approach. Mixed results for the appropriateness and acceptability of possible applications in clinical practice might reflect the early state of these applications. The theory itself, methods for network construction, and the consequences of the network theory for treatment planning and evaluation must be better understood before this approach can be responsibly applied to clinical practice. Still, the conceptualization of mental disorders as symptom networks seems to resonate with mental health professionals and might be an important component of a potential new paradigm for the diagnosis and treatment of psychopathology.

## Supplementary Materials

The Supplementary Materials contain the following items:

- Preregistration (Schumacher & Kriston, 2023S)
- Research data, code, and additional materials (Schumacher & Kriston, 2025S)

SchumacherL.
KristonL.
 (2023S). A clinical perspective on the network theory of mental disorders
[Preregistration]. PsychOpen. https://osf.io/g7zy3


SchumacherL.
KristonL.
 (2025S). Supplementary materials to "Mental health professionals’ attitudes towards the network theory of mental disorders"
[Research data, code, materials]. PsychOpen. https://osf.io/zan2q/files
10.32872/cpe.17941PMC1295530741783471

## Data Availability

The supplementary materials, the code, and the analysed data is openly available on the Open Science Framework: https://osf.io/zan2q/files

## Preregistration

The study design was preregistered on the open science framework: https://osf.io/g7zy3. We did not specify specific hypotheses for the outcome.

## Reporting Guidelines

We followed the JARS–Quant reporting guidelines for empirical research.

## Related Versions

The original (not peer-reviewed) version of this manuscript has been uploaded as a preprint on PsyArXiv: https://osf.io/preprints/psyarxiv/462cs

## References

[sp1_r1] Schumacher L. Kriston L. (2023 S). A clinical perspective on the network theory of mental disorders [Preregistration]. PsychOpen. https://osf.io/g7zy3

[sp1_r2] Schumacher L. Kriston L. (2025 S). Supplementary materials to "Mental health professionals’ attitudes towards the network theory of mental disorders" [Research data, code, materials]. PsychOpen. https://osf.io/zan2q/files 10.32872/cpe.17941 PMC12955307 41783471

